# Supplementary material for: Daylight savings time transition and the incidence of femur fractures in the older population: a nationwide registry-based study
Source: PeerJ. 2022 Aug 19;10:e13672. doi: 10.7717/peerj.13672 (PMC9394509; doi:10.7717/peerj.13672)
Supplement: Supplemental Information 1 [file peerj-10-13672-s001.html]

Skripti\_70+


# Skripti\_70+

#### Ville Ponkilainen (Topias Koukkula)

#### 5/10/2021 (update 4/2/2022)

```
knitr::opts_chunk$set(warning =  F, message=F)
library(MASS)
library(tidyverse)
library(zoo)
library(cowplot)
library(grid)
library(lubridate)
library(broom)
library(readr)
library(effects)
library(openxlsx)
library(reshape2)
library(readxl)
Sys.setlocale("LC_ALL", "en_US.UTF-8")
load("Frekvenssitaulu_70_plus.Rdata")
load("lonkat_70_plus.Rdata")
load("fig_1_data.Rdata")
```

## Preparing data set

```
pop <- read_excel("vakiluku_key.xlsx") %>% 
  mutate(IKA = as.numeric(IKA)) %>% 
  filter(IKA>=70 & year>=1997)  %>% 
  mutate(year = as.factor(year)) %>% 
  group_by(year, IKA) %>% 
  summarize(population = sum(population))
```

```
yearly_pop <- pop %>% 
  group_by(year) %>% 
  summarize(pop=sum(population))
```

```
pop_props <- pop %>% 
  left_join(yearly_pop) %>% 
  mutate(prop = population/pop) %>% 
  ungroup()
```

```
Fig1 <- lonkat_70_plus %>% 
  mutate(IKA = ifelse(IKA>=100, 100, IKA)) %>% 
  count(VUOSI, IKA)
```

```
## Preparing figure 1
Fig1_adj <- Fig1 %>% 
  mutate(year = as.factor(VUOSI)) %>% 
  left_join(pop_props) %>% 
  mutate(crude = n/population*100000,
         year=as.factor(year)) %>% 
  mutate(age_adust = crude*prop) %>% 
  filter(!is.na(age_adust)) %>% 
  group_by(year) %>% 
  summarize(adj_inc = sum(age_adust))
```

```
#"The data included a total of 110 725 femur fractures during the study period between 1997 and 2018..."
Frekvenssitaulu_70_plus %>% 
  summarize(sum(Freq))
```

```
##   sum(Freq)
## 1    110725
```

```
## ".. with an annual mean (SD) of 5 033 (308) fracture"

Frekvenssitaulu_70_plus %>% 
  group_by(vuosi) %>% 
  summarize(n = sum(Freq)) %>% 
  ungroup() %>% 
  summarize(mean = mean(n),
            sd=sd(n))
```

```
## # A tibble: 1 x 2
##    mean    sd
##   <dbl> <dbl>
## 1 5033.  308.
```

## Figure 1

```
##  Figure 1 - Yearly incidence 
#png("Fig1. Yearly variance.png", units="in", width=8, height=5, res=300)
 ggplot (data = Fig1_adj, aes(x=year,y=adj_inc)) + 
   geom_line(aes(group=1), size=1) +
    theme_classic() +
    theme(legend.position = "bottom",
          panel.grid.major.y=element_line(colour="gray50", size=0.25),
          axis.text.y = element_text(color = "grey20", size = 13),
          axis.text.x = element_text(color = "grey20", size = 13, angle=45, hjust=1),
          axis.title.y = element_text(color = "grey20", size = 16),
          axis.title.x = element_text(color = "grey20", size = 16),
          legend.text = element_text(color = "grey20", size = 14),
          strip.text.x =element_text(color = "grey20", size = 14)) +
    labs(y="Incidence per 100,000  person-years", 
         x=NULL,
         fill=NULL,
         group=NULL,
         color=NULL) +
    scale_y_continuous(limits=c(0, 1000))+
    scale_color_brewer(palette="Set2")
```

```
 #dev.off()
 
Fig1_adj %>% view
```

## Figure 2

```
### Preparing Figure 2 
Fig2 <- Frekvenssitaulu_70_plus %>% 
   mutate(viikko = as.numeric(viikko)) %>% 
   select(viikko, insidenssi) %>%
   group_by(viikko) %>%  
   summarize(n=sum(insidenssi) / 22)
```

```
## Figure 2 - weekly mean incidence
#png("Fig2. Seasonal variance.png", units="in", width=8, height=5, res=300)
Fig2 %>% 
  filter(viikko!=53) %>% 
  ggplot(aes(x=viikko,y=n)) +
  geom_line() +
  theme_classic() +
  theme(panel.grid.major.y=element_line(colour="gray50", size=0.25)) +
  annotate("segment", x = 14, xend = 14, y = 126, yend = 140, alpha=0.9, size=0.5, 
           arrow=arrow(type = "closed", length = unit(0.02, "npc"))) +
  annotate("segment", x = 44, xend = 44, y = 126, yend = 140, alpha=0.9, size=0.5, 
           arrow=arrow(type = "closed", length = unit(0.02, "npc"))) +
  labs(x="Week", 
       y="Weekly incidence", 
       colour=NULL) +
  theme(legend.position = "bottom",
        panel.grid.major.y=element_line(colour="gray50", size=0.25),
        axis.text.y = element_text(color = "grey20", size = 13),
        axis.text.x = element_text(color = "grey20", size = 13),
        axis.title.y = element_text(color = "grey20", size = 16),
        axis.title.x = element_text(color = "grey20", size = 16),
        legend.text = element_text(color = "grey20", size = 14),
        strip.text.x =element_text(color = "grey20", size = 14)) +
  scale_y_continuous(limits=c(0, 185), breaks=seq(0, 185, 25)) +
  scale_x_continuous(limits=c(0, 52), breaks=seq(0, 52, 5))
```

```
#dev.off()

Fig2 %>% view
```

### Negative binomial regression

```
# Preparing NB-regression explanatory variables
Frekvenssitaulu_70_plus <- Frekvenssitaulu_70_plus %>% 
  mutate(viikonpaiva = fct_relevel(viikonpaiva, c("Monday","Tuesday","Wednesday","Thursday","Friday","Saturday","Sunday")),
         DST_maanantai_kevt = ifelse(DST_sekvenssi %in% c(8, 29)  & DST == 1 & Vuodenaika=="Kevät", 1, 0),
         DST_kevt = ifelse(DST_uusi==1 & Vuodenaika=="Kevät", 1, 0), 
         DST_maanantai_syksy = ifelse(DST_sekvenssi %in% c(8, 29)  & DST == 1 & Vuodenaika=="Syksy", 1, 0),
         DST_syksy = ifelse(DST_uusi==1 & Vuodenaika=="Syksy", 1, 0),
         vuosi = as.factor(vuosi)) %>% 
  arrange(paivamaara, vuosi, viikko, viikonpaiva)
```

```
# Negative binomial models

## A = Monday spring
malli_maanantai_kevt <- glm.nb(insidenssi ~ DST_maanantai_kevt + as.factor(vuosi), data= Frekvenssitaulu_70_plus)
```

```
## B = Monday autumn
malli_maanantai_syksy <- glm.nb(insidenssi ~ DST_maanantai_syksy + as.factor(vuosi), data= Frekvenssitaulu_70_plus)
```

```
## C = Monday week spring
malli_viikko_kevt <- glm.nb(insidenssi ~ DST_kevt + as.factor(vuosi) + viikonpaiva + as.factor(viikko), data= Frekvenssitaulu_70_plus)
```

```
## D = Monday week autumn
malli_viikko_syksy <- glm.nb(insidenssi ~ DST_syksy + as.factor(vuosi) + viikonpaiva + as.factor(viikko), data= Frekvenssitaulu_70_plus)
```

#### Regression outputs tidy

```
A <- tidy(malli_maanantai_kevt, conf.int=T) %>% 
  filter(term=="DST_maanantai_kevt") %>% 
  transmute(IRR = exp(estimate),
            lower = exp(conf.low),
            upper = exp(conf.high))
A
```

```
## # A tibble: 1 x 3
##     IRR lower upper
##   <dbl> <dbl> <dbl>
## 1  1.08 0.952  1.22
```

```
B <- tidy(malli_maanantai_syksy, conf.int=T) %>% 
  filter(term=="DST_maanantai_syksy") %>% 
  transmute(IRR = exp(estimate),
            lower = exp(conf.low),
            upper = exp(conf.high))
B
```

```
## # A tibble: 1 x 3
##     IRR lower upper
##   <dbl> <dbl> <dbl>
## 1  1.11 0.984  1.26
```

```
C <- tidy(malli_viikko_kevt, conf.int=T) %>% 
  filter(term=="DST_kevt") %>% 
  transmute(IRR = exp(estimate),
            lower = exp(conf.low),
            upper = exp(conf.high))
C
```

```
## # A tibble: 1 x 3
##     IRR lower upper
##   <dbl> <dbl> <dbl>
## 1  1.07  1.01  1.14
```

```
D <-tidy(malli_viikko_syksy, conf.int=T) %>% 
  filter(term=="DST_syksy") %>% 
  transmute(IRR = exp(estimate),
            lower = exp(conf.low),
            upper = exp(conf.high))

D
```

```
## # A tibble: 1 x 3
##     IRR lower upper
##   <dbl> <dbl> <dbl>
## 1 0.957 0.821  1.11
```

## McFadden Pseudo R^2

```
### McFadden pseudo-R2
mallit <- data.frame(c(1 - (malli_maanantai_kevt$deviance / malli_maanantai_kevt$null.deviance), 1 - (malli_maanantai_syksy $deviance / malli_maanantai_syksy $null.deviance), 1 - (malli_viikko_kevt $deviance / malli_viikko_kevt $null.deviance), 1 - (malli_viikko_syksy $deviance / malli_viikko_syksy $null.deviance)), c("malli_maanantai_kevt", "malli_maanantai_syksy", "malli_viikko_kevt", "malli_viikko_syksy"), c(8032 - malli_maanantai_kevt$df.residual, 8032 - malli_maanantai_syksy $df.residual,  8032 - malli_viikko_kevt $df.residual, 8032 - malli_viikko_syksy $df.residual  ))
colnames(mallit) <- c("McFadden R2", "Model", "df vs. null model")
mallit
```

```
##   McFadden R2                 Model df vs. null model
## 1  0.09820158  malli_maanantai_kevt                22
## 2  0.09836929 malli_maanantai_syksy                22
## 3  0.22777645     malli_viikko_kevt                80
## 4  0.22737133    malli_viikko_syksy                80
```

## Table 1

```
## Preparing data set by age

lonkat_70_plus$IKA_luok <-cut(lonkat_70_plus$IKA, br=c(0,74,79,84,89,94,99,120))
levels(lonkat_70_plus$IKA_luok ) <- c("70-74", "75-79","80-84", "85-89","90-94", "95-99", "100+")

murtumat_n_ika <- as.data.frame(table(lonkat_70_plus$IKA_luok, substring(lonkat_70_plus$TUPVA_date_format,1,4)))

colnames(murtumat_n_ika) <- c("IKA", "Vuosi", "Freq")
murtumat_n_ika  <- reshape(murtumat_n_ika, idvar= "Vuosi", timevar="IKA", direction="wide")
colnames(murtumat_n_ika) <- c("Vuosi", levels(lonkat_70_plus$IKA_luok ))
```

```
## Sum of fractures, by age class
murtumat_n_ika_yht <-  colSums(murtumat_n_ika[,2:ncol(murtumat_n_ika)])
murtumat_n_ika_yht
```

```
## 70-74 75-79 80-84 85-89 90-94 95-99  100+ 
## 13538 21415 28848 28496 14828  3305   303
```

```
## Prob of fractures, by age class
murtumat_n_ika_prob_yht <-  prop.table(murtumat_n_ika_yht)
murtumat_n_ika_prob_yht
```

```
##       70-74       75-79       80-84       85-89       90-94       95-99 
## 0.122258044 0.193393117 0.260518545 0.257339727 0.133907688 0.029846568 
##        100+ 
## 0.002736312
```

```
## preparing data set by gender

murtumat_n_sukup <- as.data.frame(table(lonkat_70_plus$SUKUP, substring(lonkat_70_plus$TUPVA_date_format,1,4)))
murtumat_n_sukup <- murtumat_n_sukup[murtumat_n_sukup[,1] == 1 | murtumat_n_sukup[,1] == 2,]
murtumat_n_sukup[,1] <- as.character(murtumat_n_sukup[,1])
murtumat_n_sukup[,1][murtumat_n_sukup[,1]  == "1"] <- "Miehet"
murtumat_n_sukup[,1][murtumat_n_sukup[,1]  == "2"] <- "Naiset"

colnames(murtumat_n_sukup) <- c("Sukup", "Vuosi", "Freq")
murtumat_n_sukup  <- reshape(murtumat_n_sukup, idvar= "Vuosi", timevar="Sukup", direction="wide")
colnames(murtumat_n_sukup) <- c("Vuosi", "Miehet", "Naiset")
```

```
## Sum of fractures, by gender
murtumat_n_sukup_yht <- colSums(murtumat_n_sukup[,c(2,3)])
murtumat_n_sukup_yht
```

```
## Miehet Naiset 
##  28102  82631
```

```
## Prob of fractures, by gender

murtumat_n_sukup_yht_prob  <- prop.table(murtumat_n_sukup_yht)
murtumat_n_sukup_yht_prob
```

```
##    Miehet    Naiset 
## 0.2537816 0.7462184
```
